# Corner Store Retailers’ Perspectives on a Discontinued Healthy Corner Store Initiative

**DOI:** 10.1177/0272684X211004930

**Published:** 2021-04-06

**Authors:** Meghan Lynch, Marketa Graham, Krystal Taylor, Catherine L. Mah

**Affiliations:** 1 Dalla Lana School of Public Health, University of Toronto, Toronto, Ontario, Canada; 2 Chronic Disease and Injury Prevention Unit, Ottawa Public Health, Ottawa, Ontario, Canada; 3 School of Health Administration, Dalhousie University, Halifax, Nova Scotia, Canada

**Keywords:** healthy corner stores, fruits and vegetables, store owners, qualitative research

## Abstract

Making fresh fruits and vegetables (FFV) more widely available has been a prominent focus of healthy retail interventions and may have an important role in improving food access and diet quality at the population level. ‘Healthy retail’ interventions in corner/convenience stores (CS) are increasingly being adopted by public health practitioners to address the diet-related risk factors, improve food access at the community level, and change food retail environments. Private sector retailers are integral to the success of public health retailing interventions, making their perspectives and experiences critical. There is a particular need for greater evidence from retailers in settings where evaluations of these interventions have yielded null or mixed results. Through semi-structured interviews with 8 CS retailers (7 from urban settings and 1 from rural) in Ottawa, Ontario, Canada, this study aimed to describe experiences and critical factors regarding the feasibility and sustainability of a healthy CS program that was not sustained following the pilot testing phase, with a specific focus on the sale of FFV. Thematic analysis was used to analyze the interview data, which indicated that retailers faced two dominant challenges with selling FFV in CS: both relate to how these stores are embedded in the larger local and global food system. We join others in arguing that efforts and support for retail interventions aiming to increase the availability of FFV in CS need to address the structure and relations of the food system, as an upstream determinant of CS retailer interest and motivation.

Diet-related risks are the leading factor in the global burden of ill health and mortality.^1^ The retail food environment has been recognized as an important site for diet interventions given its prominent role in shaping community food access and dietary behaviour.^2^ ‘Healthy retail’ interventions have been adopted in a range of communities and businesses, typically partnering with public health. Of particular interest are convenience/corner store (CS) interventions, where public health has adopted a mixed community and economic development approach in these retail settings to target improved diet-related outcomes.^2,3^ The healthy corner store (HCS) model is a comprehensive complex population health intervention in small community retail stores, receiving growing attention in the literature.^4,5^ In most cases, HCSs are ‘conversions’ of existing traditional CSs into ones that are more health-promoting, through changes to the business model and infrastructure, food items sold in store, and other aspects of retailing practice.^6^ However, as a whole, the evidence to date on HCSs has been mixed, owing often to the substantial heterogeneity among interventions, settings and their evaluations,^7–9^ with less research focused on the barriers for healthy retail implementation.^10^ In particular, the literature is only beginning to emerge on how CS owners and managers (referred collectively as ‘retailers’ for the remainder of this manuscript) are willing and able to accept, implement, and sustain healthy retail interventions, while others are not.^11^ Only recently have researchers focused more on retailers’ roles in shaping food access within the food system, by, for example, considering the need to specifically target retailers and assess the effectiveness of interventions using both health and business-relevant outcomes.^12,13^

A variety of factors influence CS retailers’ decisions about stocking food and beverages in their stores, among other consumer products,^10,14^ which can affect their willingness to implement healthy retailing criteria.^15,16^ One of the most consistent findings to date has been retailers’ perceptions regarding a lack of customer demand for fresh fruits and vegetables (FFV).^10,17^ Making FFV more widely available and accessible has been a prominent focus of healthy retail interventions and increasing consumption may have an important role in improving diet quality and reducing diet-related risk at the population level.^1^ Yet, research with retailers has described how retailers reported stocking more unhealthy than healthy food options due to both customer demand for unhealthy foods and lack of customer interest in healthy foods.^11,18,19^ Concerns regarding lack of customer demand may be further compounded by retailers’ perceptions that FFV are not as profitable as unhealthy products^20^—reflecting the wide variation in actual and acceptable profit margins for the diverse array of product lines in a typical convenience store. These stocking challenges are further intertwined with concerns over reducing space allotted to products sold in the greatest volume in the CS: tobacco and alcohol.^11,20^ Adding to this, retailers have described previously stocking FFV but being forced to stop due to failing to sell enough FFV to cover costs, often resulting from the wastage of FFV and loss of revenue.^11^

FFV wholesale produce vendors also play an important role in the abilities of CS retailers to stock fresh produce.^10,17,21^ For example, the most common challenge reported by retailers in one HCS study was obtaining a consistent supply of FFV.^15^ Shelf-stable, pre-packaged snack food items were more likely to be directly delivered and stocked by suppliers, while FFV were often the retailers’ responsibilities, possibly due to distributors’ disinterest in dealing with CSs.^11^ One case study of a small food store retailer who began selling healthy food options attributed his store’s success to the positive relationship with his supplier.^22^ This does not appear to be the norm: interviews with local produce wholesalers in New Orleans revealed that the wholesalers did not consider CSs as profitable business opportunities, instead describing multiple issues they encountered when working with CS retailers, including CS retailers requesting multiple products in small volumes, ordering deliveries late, and cancelling deliveries.^19^ These are important challenges to consider, as without a local wholesaler, retailers were left to self-stock FFV, a practice that has been found to be burdensome and difficult for CS retailers to sustain.^16^

As this overview of past research with CS retailers demonstrates, a growing evidence base has documented both individual and food supply (practical and logistical) challenges CS retailers face in shifting their business model in a healthier direction.^23^ Understanding CS retailers’ viewpoints and attitudes toward HCS interventions can support progress towards intended health outcomes.^17^ Past research has recommended researchers further explore CS retailers’ perceptions and perspectives, particularly examining the barriers to implementation, in order to better develop HCS interventions that can meet both business and public health goals.^10,16,17^ There is a particular need for greater evidence from retailers in settings where evaluations of these interventions have yielded null or mixed results.

To add to this literature, we undertook a series of interviews with CS retailers in Ottawa, Ontario, Canada, who were approached by the city’s public health unit to participate in a pilot HCS program, from 2017–2018. One of the main aims of the Ottawa HCS initiative, *Good Food Corner Stores* (GFCS), was to increase access to FFV and other healthy staples for low-income community residents through the CS environment. Following the six-month HCS pilot program, the program was discontinued; the HCS model required significant investments to improve both processes in procuring and selling FFV and customer demand, which stakeholders were not interested in at that time.^24^ Following the completion of the pilot program, academic partners began working with the public health unit to continue to evaluate the processes and outcomes of the HCS initiative in greater depth, including more detailed qualitative research with retailers. The intent of the post-hoc evaluation was to inform future public health practice in the unit as well as promote sharing of experiences in the literature and with other jurisdictions. We undertook interviews both CS retailers who participated in HCS and those who were approached by the public health unit but did not participate. Through this sampling approach, we aimed to explore key themes for the implementation and sustainability of the HCS, specifically, as well as contextual attitudes and beliefs about selling FFV, more generally. Our focus on FFV, therefore, enabled us to understand, from the CS retailers’ perspectives, the context of the HCS pilot program.

## Background Context

The city (Ottawa, Ontario, Canada), where the HCS intervention took place had approximately 377 CS located in 108 neighbourhoods at the time of the pilot, including stores across 40 low socioeconomic status neighbourhoods. Over half of CS, 59% (221), of CS in Ottawa were independently-owned and 156 were chain-operated. Independently-owned CS were small, usually family-owned, controlled, and operated businesses that had a minimum number of employees and only a small amount of business volume. There were seven main convenience store chain operators that were larger in size with multiple locations.

The HCS initiative aimed to make it easier for community residents to purchase FFV in the CS environment, particularly in low-income neighbourhoods that did not have local access to a major grocery retailer. In 2015, the health unit staff conducted outreach to 78 CS and selected eight diverse CSs from seven neighbourhoods to participate in the HCS pilot. Out of the eight participating CSs, seven were independently owned and operated and one was chain-operated. Seven CSs were located in urban settings; one CS was located in a rural location within the city boundaries. Important to note, one participating CS was unique in that the model by which FFV were supplied and marketed within this CS was developed and funded by a university business student group. Seven CSs completed the pilot; one CS closed due to the owner’s illness.

Retailers agreed to a six-month pilot and committed to following GFCS criteria (Table 1). None of the CS met the HCS criteria prior to the pilot; however, six of the eight stores were selling some quantities of FFV. Therefore, for those CSs that sold FFV before the pilot, the aim of the pilot was to continue FFV and support retailers in attaining HCS criteria for other staple foods, as well as to learn from their current practices around stocking FFV. During the pilot, public health staff followed up with CS retailers weekly for the first month and at least monthly thereafter. The public health unit also provided branding, promotion, resources (including baskets and shelving materials for displaying FFV), and links to community support.

**Table 1.** Good Food Corner Store Criteria.

At least 3 different fresh vegetables (e.g. potatoes, carrots, tomatoes, onions)
At least 3 different fresh fruits (e.g. bananas, apples, oranges, lemons)
At least 2 whole grain products (e.g. whole grain bread, oatmeal, brown rice)
At least 2 lower fat milk products (e.g. milk, yogurt 2% or less)
At least 2 different meat alternatives (e.g. eggs, beans, canned fish, tofu)
Display Good Food Corner Store marketing materials within the store
Actively participate in Good Food Corner Store project activities, including, store assessments, implementation, and evaluation
Document and share sales orders of foods involved in Good Food Corner Store
Maintain a clean premise and adhere to food safety standards

## Methods

### Data Collection

We developed a semi-structured interview guide (Supplementary File A) that was based on previous HCS literature and Diffusion of Innovations Theory^25^ to ensure consistency while also allowing for flexibility to discuss topics the retailers themselves raised. Between January 2019 and May 2019 interviews were conducted in-person in each of the participant’s CS during regular work hours, except for one which was conducted over the phone due to participant preference. Conducting interviews during regular work hours enabled both the participants to not have to take any time off work for the interviews, and the interviewer to collect fieldwork notes through direct observation.

All interviews were conducted by the same researcher (Author Initials), a female postdoctoral fellow in public health from the University of Toronto, whose work has focused on analyzing policies and programs focused on improving the food environment, with over ten years of experience conducting and analyzing qualitative research interviews. [Author Initials] had not participated in any aspect of the HCS pilot and as such had no relationship with any of the study participants prior to study commencement. Participants were introduced to [Author Initials] as a researcher who was interested in learning more about their experiences with the HCS and/or selling fruits and vegetables in CSs.

During and following each interview, [Author Initials] took field notes on the physical environment of the stores, the presence of customers, and their interactions with the CS retailers. Interviews were between 22 and 68 minutes and lasted on average 41 minutes and all participants were interviewed once.

### Data Analysis

Interviews were digitally recorded with participants’ consent and transcribed verbatim. Potentially identifying details were omitted, and all participants’ names were given numbers to ensure confidentiality and anonymity. Transcripts and field notes were then imported into the qualitative data analysis software NVivo for analysis. Transcripts were not returned to participants for comments or corrections due to participants’ expressed lack of time (member checking was not conducted for the same reason).

The interview data were analyzed by the first author using an approach of constant comparison that entailed summarizing and classifying the data and then relating it to previous literature.^26,27^ The second, third, and fourth authors assisted in refining the coding structure and/or contributed to the analytic process through regular discussions with the first author, [Author Initials].

Transcript reading and preliminary analysis were conducted sequentially. By drawing concepts directly from the data and also having an idea of what we were looking for based on related literature, the analysis was not entirely inductive or deductive, but a combination of both. Initial codes were identified challenges that participants described as preventing them from procuring and selling FFV. Each type of challenge was labelled, and, when appropriate, different challenges were combined into one broad category.^28^ Initial codes were applied to later data, new codes developed as new themes emerged, and some initial codes were revised. Next, related codes were then grouped together under the main themes. As patterns in the data developed, all authors worked as a team to develop concepts that could explain them. Throughout the analysis, we found it important to spend a prolonged period of time immersed in the data, as the more familiar we became with the data, the better we were able to generate codes and themes and identify patterns along with looking for disconfirming instances and alternative explanations. Resulting from this analysis, the main themes of the data are presented below. Examples and quotations were selected because they are typical of the themes identified. Quotations are presented with minor speech hesitations and grammatical errors corrected to facilitate readability.^27^

## Results

### Participants

This study was approved by the University of Toronto and Ottawa Public Health’s ethics review boards and all participants provided voluntary informed written or verbal consent. To be included, participants must have been previously approached by the public health unit to participate in the HCS in Ottawa, Ontario, Canada.

CS retailers were contacted by telephone between December 2018 and February 2019 and provided with information on the study and asked to participate in a one-on-one interview. We approached 15 CS retailers to participate in the study (Table 2) and eight retailers (six store retailers who participated in the HCS program and two who were approached during recruitment but did not participate in the HCS) agreed to participate. Two retailers who chose to not participate in the HCS were included in the interviews because we believed their perceptions and experiences would strengthen our understanding of contextual features surrounding implementation of the HCS pilot program.

**Table 2.** Corner Store and Retailer Characteristics.

Corner store	Urban or rural setting	Independent or franchise	Corner store owner or manager	Participated in HCS pilot	Participated in interview
CS 1	Urban	Independent	Owner	No – CS met HCS criteria prior to pilot program	Yes
CS 2	Urban	Independent	Owner	Yes	Yes
CS 3	Urban	Independent	Owner	Yes	Yes
CS 4	Urban	Franchise	Manager	Yes – 1 location	Yes
CS 5	Rural	Independent	Owner	Yes – Owner sold the CS following the HCS pilot program	Yes – phone interview
CS 6	Urban	Independent	Owner	Yes	Yes
CS 7	Urban	Independent	Owner	No	Yes
CS 8	Urban	Independent	Owner	Yes – Participated in the program run independent of the HCS	Yes
CS 9	Urban	Independent	Owner	Yes	No – CS closed
CS 10	Urban+ Rural	Franchise	Manager	No	No
CS 11	Rural	Independent	Owner	No	No
CS 12	Urban	Independent	Owner	No	No
CS 13	Urban	Independent	Owner	No	No
CS 14	Urban	Independent	Owner	No	No
CS 15	Urban	Independent	Owner	No	No

Among retailers that agreed to participate in an interview, seven of the interviewees were CS owners and one was a manager of a number of franchised chain CS. Six of the eight participating retailers identified as men. Five of the CS retailers had participated in the HCS pilot program described earlier. One retailer did not participate in the program because the CS was already selling the minimum quantity of FFV prior to the HCS intervention. One retailer was approached to participate but declined, and one retailer participated in the co-located program (this store had all of the FFV purchased, delivered, and waste collected by a university program that was being run in conjunction with the HCS pilot program). Only one store was located in a rural setting, but unless otherwise noted, we did not observe any differences in the responses between the urban and rural CS retailers.

### Results Overview

We found that retailers faced two dominant challenges with selling FFV in CSs: both related to how these stores are embedded in the larger local and global food supply. In the following section, we describe these two major themes. The first theme describes how the local food supply and retail sector was not conducive for CS to sell FFV and resulted in CS retailers perceiving themselves to be at a disadvantage when selling FFV compared to other food retail outlets. The second theme reflects the struggle retailers expressed in wanting to provide a service to their customers with healthy foods but framed this as a trade-off against needing their CS to be financially viable. Together, these findings indicated a range of practical and relational challenges faced by retailers in adopting healthier practice with local contextual factors. We further interpreted these findings in light of the HCS program not being sustained following the pilot program, and how HCS interventions need to be adapted to different local contexts.^2^

### Theme 1: Local Retail Food System Unfriendly for CS to Sell FFV

This first theme encompasses one of the most frequent points that came up in the CS retailer interviews, when they were asked to describe their experiences with being part of the HCS pilot program, and for those not participating in the pilot, their experiences with purchasing FFV to sell in their CS. Retailers described how they felt CS were at a disadvantage compared to other competing Ottawa food retail outlets in terms of purchasing, stocking, and retailing FFV.

#### Time Spent Acquiring FFV

CS retailers detailed the challenges and frustrations with acquiring and selling the variety of FFV required to meet HCS criteria, describing how they personally spent additional time shopping for FFV mainly through retail sources—and at retail prices—in order to meet HCS criteria. Retailers addressed this imperative by searching through weekly grocery store sales flyers to determine which grocery store was selling which FFV at the lowest price that week and then driving throughout the city to different grocery stores to purchase different FFV. Some even described needing to repeat this process of shopping for FFV daily, before opening their CS. This shopping process was not unique to FFV; retailers explained only a limited number of products were delivered to their CS. Consequently, they reported spending an inordinate and excess amount of travel, labour, and time stocking their CS. CS 7, a retailer who declined to participate in the HCS, articulated: “Cigarettes are delivered. For everything else sold, I am physically going, collecting, buying, shopping, carrying, marking, and then putting it on the shelves. Oh, and actually chips are delivered. Chips and cigarettes. That’s it.”

#### Unpredictability of FFV Prices

Acquiring FFV to sell in their CS presented challenges unique to FFV due to what was described as the unpredictability of FFV pricing. All CS retailers described how they purchased FFV at retail from other retail food outlets in the city, including grocery stores, wholesale clubs, and multipurpose stores, to then resell in their CS. They described how because the retail prices of FFV changed week to week in the grocery stores, they were left being unable to estimated how much it was going to cost them each week to acquire FFV for their CS. This volatility of FFV pricing was typically juxtaposed with other products sold in their CS, which were described as having more stable prices. CS retailers often contrasted their challenges with the perceived ease with which they believed Ottawa-area grocery stores were able to acquire and sell FFV at lower prices than CS. A few described their attempts to offer products at prices lower than grocery store prices. However, most explained that selling products, especially FFV, at lower prices than grocery stores was impossible for them to do without losing money.

#### Weather

Ottawa’s winters further contributed to these challenges. CS 1, who was not part of the HCS pilot, detailed the unpredictability of FFV prices: "This is the worst year I have seen this time of year [winter] for produce.” Second, FFV’s perishability made them distinct from other CS products, as they resulted in wastage (and financial loss) if turnover was inadequate. When faced with FFV waste, CS retailers described needing to forgo profits and either take home the produce for personal consumption, or simply give customers FFV free of charge. Here, again, Ottawa’s winters exacerbated this challenge, as retailers described encountering more waste in colder weather. CS 2 described how customer demand for FFV also decreased in the winter because “it just feels different. I feel like when you are in Ottawa, like the winter, everyone is just kind of hibernating at that point.”

#### Distribution Barriers

Given the struggles described above in retailing FFV, it was not a surprise that needing a FFV wholesale produce vendor was a major concern for many. Indeed, when asked what they believed was needed to acquire the variety of FFV required for the HCS pilot, retailers commonly cited needing to alleviate FFV distribution barriers. CS 2, for instance, definitely stated: “You need a good supplier that can give you phenomenal prices." This sentiment was echoed throughout all the interviews. Adding to the supply challenges, retailers additionally explained how they could not afford FFV delivery to their CS. The sole CS retailer – CS 8 – who was getting FFV delivered to her CS was only able to do so because she was the retailer who was part of the initiative run out of the university that supplied FFV to her store. Many believed all the CS in the HCS pilot program should have been provided to CS with distribution (purchasing/ordering and delivery), with some expressing frustrations with the HCS pilot because it did not. Other retailers, such as CS 11 and CS 7, declined to participate in the HCS because the program would not be providing them with FFV supply. However, CS 4, a manager of multiple franchised CSs in the city provided insight into how he understood why delivery was simply not feasible from the wholesalers’ perspective: “Another huge problem is getting delivery. Because to have a truck deliver you a case of bananas, six onions, and you know a dozen apples it just doesn’t pay. It doesn’t pay them to do it.”

### Theme 2: Customers Health Versus Financial Viability

The second theme reflected the tensions retailers expressed regarding their interest in selling FFV to improve customers’ health which was seen as a countervailing trade-off to needing their CS to be financially viable businesses.

#### Customers Health

Retailers appeared to have amicable relationships with their customers: During the interviews, many retailers were observed greeting their customers by their names, engaging in friendly conversations with them, and often appearing to know what product(s) the customer was there to purchase. Being able to provide their customers with affordable healthy food options was frequently mentioned as a reason behind selling FFV, regardless of whether they participated or declined to participate in the HCS pilot program. The majority described their customers as being interested in eating healthy; for example, CS 8 described her customers interest in purchasing FFV as: “I think people really like to eat healthy…If it [motions to FFV] is more readily available and accessible to communities, I think people will buy it.” Retailers additionally expressed how they understood the financial struggles their customers faced when shopping for FFV, explaining how their customers could only purchase FFV from stores that sold them at “affordable prices,” which meant prices that were similar to local grocery stores. CS 3 articulated the struggles his CS’s low-income customers faced when purchasing FFV:

I find money is the main thing … They don’t have money. Most of the time, people who eat bananas, oranges, and apples, those people are middle class, and they have money. For young people, like students, money is tight. They can’t spend extra money on food.

Consequently, because retailers needed to sell FFV in their CS at higher prices than grocery stores, they believed their customers would seldomly purchase FFV from the CS. They described how their customers purchased their weekly groceries, including FFV, from grocery stores and would only purchase FFV from CSs in instances where they forgot to purchase a FFV from the grocery store. This belief that customers would only purchase FFV from a CS only if they, as a few described, “got stuck,”—an exceptional circumstance rather than a regular shopping behaviour—resulted in CS owners being hesitant to carry the varieties of FFV that was a requirement of the HCS program.

#### Financial Viability

But while all expressed interest in providing their communities with healthy foods, outweighing this interest was the importance of their CSs being financially viable. When asked to describe why they wanted to participate in the HCS or why they chose to sell FFV in their CSs, the main reason voiced by retailers was they viewed selling FFV as a way to increase revenue for the CS, such as by preventing customers from going “somewhere else” for FFV. CS 1 may have stated it best, when, after describing the importance of his CSs role in the community, he further reflected how he carried FFV because: “I don’t have a big super market nearby. Say, one opened? I would still sell cigarettes, and candy, but I wouldn’t sell (motions to vegetables)  … because we can’t compete.” In line with this was how CS owners often expressed the importance of giving customers the “choice” between purchasing unhealthy and healthy foods from the CS; all retailers were disinterested in eliminating unhealthy choices. FFV were occasionally described negatively because they were believed to crowd out more profitable (and unhealthy) products by a number involved in the HCS pilot. During store observations, FFV were often noted as being sold at the back of the CS, where, retailers, such as CS 5 commented, customers often do not even “know we have that stuff [FFV] in the back." CS 4, a manager of several franchised CSs in the city, when asked about moving the FFV to the front of his CS, where it would be more visible to customers, replied: "You know this should be, if you really want to do it, it should be at the front of the store.” When asked if he was not permitted to change the configuration of the store to highlight FFV he replied:

If I wanted to, I could. Just move everything down. But there is not that much money in that [FFV] … It is just that 50% [of the CS profits] is tobacco, 50% is everything else. So that includes soft drinks, chips, bread, and milk, so FFV is just a minor piece. How much effort are you going to put in? And what you are going to get back in return?

In a similar vein, CS retailers described investing money in changes to their CSs if they perceived the changes would result in customers purchasing more items from the CS. For example, spending money on making electrical and flooring upgrades and decorating CS for holidays; CS 2 described such investments as “just little things to increase more business. More sales, right?" Similarly, many described modifying how they initially sold FFV to increase revenue, such as by breaking down large packages of grapes into smaller containers for customers to purchase, after customers requested the change.

## Discussion

The findings from this case study provided necessary descriptions of how a sample of Ottawa CS retailers, both those who participated in a HCS pilot program and those that did not, struggled in terms of both supply and demand for FFV in CS. Regarding what can be gleaned from this study to aid the development of HCS interventions that are sustainable for CS retailers, of particular interest were: first, CS retailers’ descriptions of the challenges they faced acquiring FFV without the express support and commitment of a FFV wholesale produce vendor. Second, their perceptions of the relational aspects of customer service in a community intertwined with concerns over financial viability. As we discuss in this section, our findings contribute to HCS research showing the need for public health practitioners to focus on reducing and mitigating the barriers facing retailers in operating their stores outright, in terms of being part of an overall retail food system, as opposed to seeking out retailers who are motivated to improve community health through encouraging healthy food purchases to participate in HCS interventions.^6,22^

The first theme described how CS retailers perceived CS to be at a disadvantage compared to Ottawa’s other retail food outlets. One of the main challenges described involved apprehensions about acquiring and selling FFV. This finding was in agreement with past research with CS retailers that reported retailers’ concerns with selling FFV centred around FFV being a high-risk investment that typically resulted in waste and financial losses.^10,11,17,20^ CS retailers explained how seasonal considerations (challenges in acquiring FFV and fewer customers due to weather) made selling FFV even more challenging in the winter months in this city; these local considerations underline the importance of investigating specific local barriers prior to any HCS intervention. To help combat these challenges, CS retailers described FFV wholesale produce vendors as the integral piece they needed to sell FFV in CS, a piece that was not part of the HCS pilot program. Without a FFV supplier and due to the perishability of FFV, they described their challenges with the HCS pilot, including the amount of time dedicated to acquiring FFV and losing money on FFV waste, findings that again echo other HCS evaluative research.^10,15^

The second theme described how all CS retailers in our study, both those who participated in the HCS pilot and those who declined, expressed both interest in selling FFV in their stores and concern for their customers health. Many knew customers by name, knew the products they typically purchased, and expressed understanding the challenges their customers faced in purchasing FFV from a CS. In this way, our interviews did not replicate important aspects of two recent reviews on HCS^10,16^ that concluded that in most HCS interventions, CS retailers perceived their customers to be disinterested in improving dietary behaviors and overwhelmingly consumer tastes were perceived to favor unhealthy foods and beverages. We also did not replicate research that contrasted retailers who expressed interest in a HCS intervention and those who were disinterested have found a common reason for disinterest was due to a perceived lack of customer demand for FFV.^11,19^ CS retailers in our study believed their customers *were* interested in purchasing FFV but were unable to for financial reasons. Ultimately, however, retailers were more concerned about the financial viability of their CSs, and perceived HCS criteria required them to acquire more FFV than they believed would be economically sustainable.

Together, these two themes point to the importance of considering the overall retail food system as the crucial component for successful HCS. While our findings do not dispute that CS retailers with amicable relationships with customers may be more willing to stock healthy foods,^17^ we contend that a supportive retail food system may be the more important piece in HCS interventions, and encourage future research to continue to investigate HCS interventions that present null or mixed results. As others have noted, we observed how CS retailers’ interest in selling FFV alone was not sufficient for them to sustain selling FFV in their CS.^29^ These insights into the challenges of the larger retail food system within which CSs are situated are being increasingly emphasized in the HCS literature.^2,10^ Based on our findings, we agree with past researchers that increasing FFVs in CS may require a systematic change in the retail food supply chain before HCS interventions can be sustainable,^16,19^ such as by developing more enabling policies in the context where HCS interventions are designed and delivered and focusing on how policies impacted retailers’ behaviours.

## Conclusion

The purpose of this study was to describe CS retailers’ experiences regarding the feasibility and sustainability of a HCS program that was not sustained following the pilot program phase, with a specific focus on the sale of FFV. In spite of the diverse sample of CS retailers included in our study, we found they experienced common challenges with selling FFV in the city of Ottawa. We join others in arguing that efforts and support for retail interventions aiming to increase the availability of FFV in CSs need to address the structure and relations of the food supply, as an upstream determinant of CS retailer interest and motivation. In particular, we stress the importance of future research targeting wholesalers in order to improve our understanding of how to develop enabling policies for retail stores.^30,31^

We note that the generalizability of our results is limited by the small sample size within one city. One of the unique contributions of this study was that the academic partners did not join until after the HCS intervention was completed by the city’s public health unit. However, this meant that CS retailers who were recruited to participate in the HCS pilot were selected by the public health unit. Additionally, the method of semi-structured interviewing resulted in CS retailers having more control over the interview topics covered, resulting in variability amongst the interviews. Nonetheless, our findings in relation to the challenges in acquiring and selling FFV provides additional context for understanding the food retail system from the CS retailers’ perspectives. By providing this perspective, we emphasized the complex challenges of implementing HCSs in settings where the larger retail food system was not effectively set up for CS to participate in healthy retailing.

## Supplemental Material

sj-pdf-1-qch-10.1177_0272684X211004930 - Supplemental material for Corner Store Retailers’ Perspectives on a Discontinued Healthy Corner Store Initiative Supplemental material, sj-pdf-1-qch-10.1177_0272684X211004930 for Corner Store Retailers’ Perspectives on a Discontinued Healthy Corner Store Initiative by Meghan Lynch, Marketa Graham, Krystal Taylor and Catherine L. Mah in International Quarterly of Community Health Education
