# Supplementary material for: Corner Store Retailers’ Perspectives on a Discontinued Healthy Corner Store Initiative
Source: Community Health Equity Res Policy. 2021 Apr 6;43(4):421–9. doi: 10.1177/0272684X211004930 (PMC10240627; doi:10.1177/0272684X211004930)
Supplement: sj-pdf-1-qch-10.1177_0272684X211004930 - Supplemental material for Corner Store Retailers’ Perspectives on a Discontinued Healthy Corner Store Initiative [file sj-pdf-1-qch-10.1177_0272684X211004930.pdf]

UNIVERSITY OF TORONTO

Semi-Structured Interview Guide – Store Owners

<u>Introduction</u>

a) Can you first tell me how you became involved in owning a corner store?
b) Can you describe what a typical day in your store is like?

<u>Innovation (GFCS model)</u>

First, we'd like you to think back to when you first heard about the Good Food Corner Stores project.

a) Can you remember how you first heard about the GFCS project? Had you heard about similar healthy eating projects before?
b) Do you remember what you thought about it? Prompts: Did you have any questions? Concerns?
c) Can you remember why you decided to try it out in your store? Prompts: What went into decision-making process? Google it? Talk to friends or family? Talk to other store owners?
d) Did the GFCS project replace anything else you had in your store at the time? Prompts: Any displays? Incentives/Deals/Sales bins/displays?

Now, we'd like you to think about having the Good Food Corner Stores project in your store.

a) Can you remember what it was like to have the GFCS in your store? What was it like at the beginning? Prompts: Did you need any help setting it up?
b) How did the GFCS project fit in with the rest of your store?
c) Can you remember how customers reacted? Did they talk to you about it? Did their reactions change over time?
d) Did you talk about the GFCS project with anyone else? Prompts: Family? Friends? Store owners?
e) Were there things you needed to change about the GFCS project to fit your store?
f) Have you kept the GFCS project in your store?

g) If not:

Can you tell me about why GFCS didn't work in your store?
Did you keep any parts of it?
Did you replace it with anything else? Or go back to how your store was before GFCS?
Would you consider trying a healthy food project again?

h) If still:
Can you describe why you decided to keep it?
Have you had any challenges or issues with it?

Social System

a) Can you describe your store for me?
b) Can you tell me about the customers who come to your store? Are there customers who you see everyday or every week?
c) Have you talked with customers about what sorts of foods they would like to buy from your store? Prompts: Fruits, vegetables, snacks
d) How do you think the GFCS program fits into the needs of your community?
e) Do customers in your store have certain cultural/ethnic food habits that need to be considered?
f) Do you know anyone else who owns a corner store? Do you talk with them?

Personal Characteristics

a) Has your store ever participated in a program like the GFCS before? How did it work out? Prompts: Physical size of your store? Customer preferences? Lack of materials?
b) How do you feel about encouraging customers to buy healthier foods in corner stores?
Does it work with certain foods but not others? Prompts: Fruits/Vegetables? Dairy? Snacks?
What do you think 'gets in the way' of people eating healthy foods? Prompts: Customer preferences? Taste? Convenience? Cost?
c) Are there other ways of selling foods that you have found work best in your store??
d) If you could develop a program to encourage your community to eat healthier what would it look like?

Demographics

Age:
Gender:
Rent or Own Store:
Year of Owning Corner Store:
